# Genetic analysis and molecular detection of resistance to chlorpyrifos mediated by the A216S substitution in acetylcholinesterase‐1 in the plant bug *Apolygus lucorum*

**DOI:** 10.1111/1744-7917.12744

**Published:** 2020-01-16

**Authors:** Kai‐Ran Zuo, Yi‐Hua Yang, Yi‐Dong Wu, Shu‐Wen Wu

**Affiliations:** ^1^ College of Plant Protection Nanjing Agricultural University Nanjing China

**Keywords:** acetylcholinesterase‐1, *Apolygus lucorum*, chlorpyrifos resistance, inheritance

## Abstract

The green plant bug *Apolygus lucorum* is a major pest of *Bacillus thuringiensis* cotton in China. Previously, we reported that chlorpyrifos resistance in a laboratory‐selected strain of *A. lucorum* (BZ‐R) is associated with the homozygosis of an allele in the *ace‐1* gene encoding an alanine to serine substitution at position 216 of acetylcholinesterase‐1. Here we describe the results of crosses between the resistant BZ‐R strain (41‐fold to chlorpyrifos) and the unselected susceptible BZ‐S strain homozygous for the wild type alanine allele at position 216. Resistance to chlorpyrifos was inherited as a semi‐dominant trait mainly controlled by a single autosomal gene and co‐segregates strongly but not completely with the serine substitution in *ace‐1*. Synergism bioassays and enzyme assays showed that minor contributions to resistance are also made by enhanced cytochrome P450 and carboxylesterase activities. A survey of 25 field populations from five Chinese provinces showed strong positive correlations between 50% lethal concentration against chlorpyrifos and S216 allele and genotype frequencies, although the most tolerant populations still only show 40%–50% S216 allele frequencies. The results above provide important information for designing effective resistance monitoring and management strategies for *A. lucorum* in China.

## Introduction

*Apolygus lucorum* (Meyer‐Dür) (Heteroptera: Miridae) is a polyphagous agricultural pest which is known to damage over 150 host plant species, including cotton, vegetables and fruit trees in many parts of the world (Wheeler, 2001; Lu *et al*., 2008). It was historically considered a minor pest of cotton and other crops in China but has become more problematic on cotton over time as a consequence of the reduction in insecticide use associated with the widespread planting of *Bacillus thuringiensis* (Bt) cotton (Lu *et al*., 2008; Wu *et al*., 2008; Bergé & Ricroch, 2010).

Organophosphate (OP) and pyrethroid insecticides are now widely used to control *A. lucorum* and other mirids in Chinese cotton fields (Lu *et al*., 2010), but their extensive use has resulted in the evolution of resistance to both classes of chemistry in some heavily sprayed populations (Liu *et al*., 2015; Zhen & Gao, 2016). As the target of OPs, acetylcholinesterases play an important role in chlorpyrifos resistance in many insect pests (Russell *et al*., 2004; Oakeshott *et al*., 2005). Previous studies have found a correlation between chlorpyrifos resistance and an alanine to serine mutation (equivalent to A201S in *Torpedo californica ace*) in acetylcholinesterase‐1 (*ace‐1*) in one laboratory‐selected and three field‐derived *A. lucorum* populations from China (Wu *et al*., 2015; Zhen *et al*., 2016). This substitution is orthologous to the target site OP resistance mutation that has been found in several other insects, including *Aphis gossypii*, *Plutella xylostella* and *Culex pipiens quinquefasciatus* (Li & Han, 2004; Lee *et al*., 2007; Zhao *et al*., 2014).

The goal of the current work is to assess the extent to which the A216S substitution in acetylcholinesterase‐1 explains chlorpyrifos resistance in *A. lucorum*. We address this principally by determining its genetic correlation with resistance in both laboratory crossing experiments and an extensive survey of 25 field populations drawn from five provinces in China. We also present synergist bioassay and enzyme assay results assessing the contribution to resistance by metabolic enzymes. Our results provide essential information for the design of effective resistance management strategies for this pest.

## Materials and methods

### Insect strains

The parent strain for our laboratory crosses was BZ, which was originally collected from Binzhou, in the Shandong Province of China during July 2011 (Liu *et al*., 2015). The susceptible strain BZ‐S was derived from BZ and is homozygous for the Ala216 residue at *ace‐1* locus (Wu *et al*., 2015). The version of the resistant derivative strain BZ‐R used in our previous paper (Wu *et al*., 2015) was established from BZ by continuous selection with chlorpyrifos for 18 generations at 50%–70% mortality. At that point its resistance had reached 21‐fold compared to BZ‐S. The current study was carried out after a further 13 generations of such selection, when its resistance had risen to 41‐fold compared to BZ‐S.

The 25 field populations used in the geographic survey were collected from cotton, vegetables and fruit trees in five geographical regions of China from 2014 to 2018 (Table 1 for details). The sampling sites were from Binzhou in Shandong province (SD‐BZ), Cangzhou in Hebei province (HB‐CZ), Anyang in Henan province (HN‐AY), Yancheng in Jiangsu province (JS‐YC) and Chuzhou in Anhui province (AH‐CZ). The first laboratory generation of progeny from these collections was used in the bioassays and *ace‐1* genotyping described below.

**Table 1:** Collection information of *Apolygus lucorum* field populations from China

Population	Collection area	Collection time	Main host plant
SD‐BZ	Binzhou, Shandong province	June, 2014–2018	*Zizyphus jujuba*, *Gossypium hirsutum*
HB‐CZ	Cangzhou, Hebei province	June, 2014–2018	*Zizyphus jujuba*
HN‐AY	Anyang, Henan province	August, 2014–2018	*Gossypium hirsutum*
JS‐YC	Yancheng, Jiangsu province	June, 2014–2018	*Vicia villosa*
AH‐CZ	Chuzhou, Anhui province	May, 2014–2018	*Medicago minima*, *Artemisia lavandulaefolia*

The laboratory strain SLF used as a susceptible control in some of the assays below was provided by the Langfang Experimental Station of the Chinese Academy of Agricultural Sciences, and is homozygous for the Ala216 residue at *ace‐1* locus (Wu *et al*., 2015).

As per Wu *et al*. (2015), all strains were reared on fresh green beans at 26 ± 1 °C and 65% relative humidity (RH) with a 15 : 9 L : D photoperiod, and adults were fed with 5% sucrose solution.

### Bioassays

A glass vial bioassay adapted from Snodgrass (1996) was used to determine lethal concentration at 50% (LC_50_) against technical grade chlorpyrifos (95% active ingredient) obtained from Hubei Xianlong Chemical Industry (Xiantao, Hubei Province, China). In each test, 0.2 mL of chlorpyrifos diluted in acetone was put into a 20 mL glass scintillation vial, which was then rolled to generate an even layer of insecticide/acetone on its inner surface. Control vials were treated with acetone alone. Five 10‐day‐old adults were then put into each vial, supplied with a piece of green bean as food, and the vial sealed with a cotton ball. Five replicate vials were set up for each chlorpyrifos concentration in each experiment. Vials were held under the same conditions as for general rearing (above) and mortality recorded after 48 h. Adults were considered dead if they were unable to move when prodded with a needle. Probit analyses were used to estimate LC_50_ values and the 95% fiducial limits of the LC_50_ estimates.

### Inheritance of resistance

BZ‐R males were crossed with virgin BZ‐S females and vice versa. Because resistance was found to be semi‐dominant, F_1_ females were then backcrossed to BZ‐S males. About 50 adults of each sex were used in each of the crosses. Responses to chlorpyrifos of BZ‐R, BZ‐S, and F_1_ hybrids from the two reciprocal crosses and the backcross progeny were determined using the bioassay above. The degree of dominance (D) was estimated according to Stone (1968) and the number of loci conferring resistance was assessed using the direct test of a one‐locus model (Georghiou, 1969; Tabashnik, 1991).

**Fig. 1:**
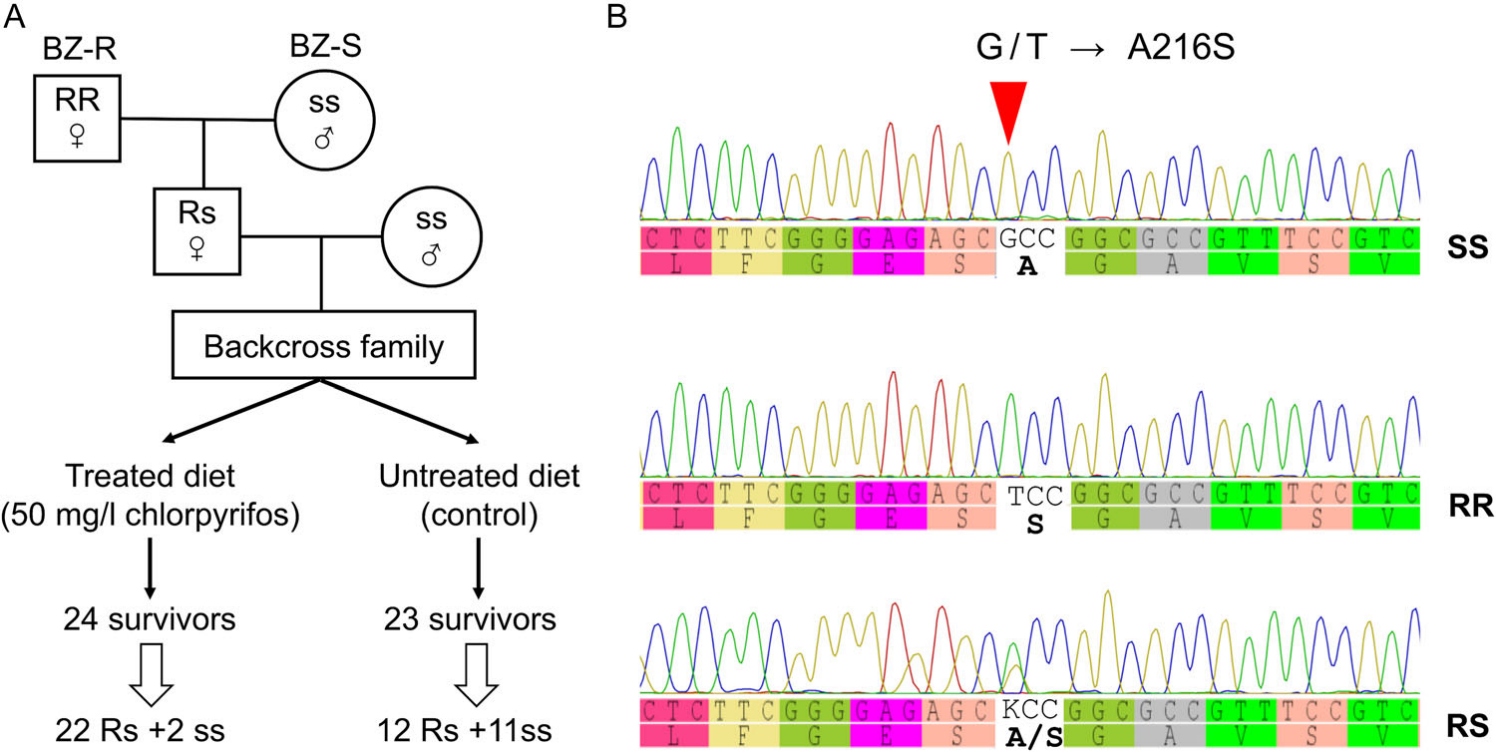
Linkage analysis of chlorpyrifos resistance with the A216S mutation of *ace‐1* in the BZ‐R strain. (A) Schematic backcross program for linkage analysis of chlorpyrifos resistance. (B) Representative chromatograms of direct sequencing of PCR products of *ace‐1*. The red triangle indicates the mutated base (G to T). SS: the wild type homozygote (216Ala/Ala); RR: the mutant homozygote (216 Ser/Ser); RS: the mutant heterozygote (216 Ala/Ser).

A backcrossing program was also used to test whether chlorpyrifos resistance was genetically linked with the *ace‐1* locus (Fig. 1). Fifty BZ‐R females were mass‐crossed with 50 BZ‐S males and 50 F_1_ females were then mass‐crossed with 50 BZ‐S males. Some of the backcross progeny were treated with 50 mg/L (about the LC_99_ of BZ‐S) of chlorpyrifos and others were kept as untreated controls. Survivors of the chlorpyrifos treatment and a similar number of controls were frozen at −20 °C for *ace‐1* genotyping.

### Detection of the A216S mutation of ace‐1

Genomic DNAs were extracted from individual adults of the wild/caught strains and backcross progeny above with an AxyPrep™ Multisource Genomic DNA Miniprep Kit (Axygen Biosciences, Union City, CA, USA) according to the manufacturer's protocol. A pair of gene‐specific primers (forward: 5ʹ‐CAGTGGGTCAGGAACAACA‐3ʹ, reverse: 5ʹ‐GCTCAGCGGTGACAGGAG‐3ʹ) was designed and used to amplify a 114 bp fragment flanking the mutation site (A216S) of ace‐1. The PCR reaction mix consisted of 12.5 *µ*L of 2× GC Buffer I (Takara, Kusatsu, Japan), 1 *µ*L of each primer (10 *µ*mol/L), 1 *µ*L of deoxynucleotide triphosphate mixture (10 mmol/L), 1 *µ*L template genomic DNA, 8.3 *µ*L sterile distilled water, and 0.2 *µ*L LA *Taq* DNA polymerase (Takara) in a final volume of 25 *µ*L. The amplification protocol was 94 °C for 3 min, then 35 cycles of 94 °C for 30 s, 60 °C for 30 s, and 72 °C for 30 s, followed by one cycle of 72 °C for 7 min. PCR products were sequenced directly with the forward primer above.

### Testing for metabolic resistance

The methods of Wu *et al*. (2015) were used to bioassay adults against chlorpyrifos in the presence of various insecticide synergists, specifically piperonyl butoxide (PBO), S,S,S‐tributyl phosphorotrithioate (DEF) and diethyl maleate (DEM), which inhibit or deplete cytochrome P450 monoxygenase (P450), carboxylesterase (CCE) and glutathione S‐transferase (GST) enzymes, respectively. P450, CCE and GST enzyme assays were also carried out on extracts of adult *A. lucorum* as per Wu *et al*. (2015).

### Data analysis software

Dose‐mortality data were analyzed via Probit analysis using POLO Plus 2.0 (LeOra Software, Berkeley, CA, USA). Correlations between chlorpyrifos LC_50_ and S216 allele and genotype frequencies of *ace‐1* gene were calculated using the IBM SPSS 19.0 Statistics software package.

## Results

### Inheritance of chlorpyrifos resistance

**Fig. 2:**
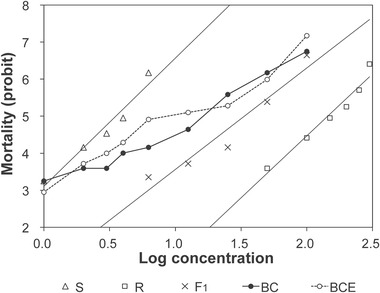
Toxicological responses to chlorpyrifos of adults from the susceptible strain BZ‐S (S), the resistant strain BZ‐R (R), F_1_ progeny (R♀ × S♂), and backcross progeny (F_1_♀ × S♂). The backcross curve (BC) was plotted using the observed mortality at each concentration. The BCE line was plotted using expected mortality values for backcross progeny calculated from a single locus model. Expected % mortality of the backcross progeny at concentration *x* = (% mortality of F_1_ at *x* + % mortality of BZ‐S at *x*) × 0.5.

Probit analyses of the dose–response curves showed the LC_50_ for chlorpyrifos of the BZ‐R strain was 41‐fold higher than that of the BZ‐S strain (Table 2). The LC_50_ values of F_1_ from the two reciprocal crosses were similar to one another (36.2 and 33.2 mg/L, with overlapping 95% fiducial limits) and intermediate between the two parents, although they were closer to the BZ‐R than BZ‐S value (dominance values of 0.24 and 0.19; Fig. 2, Table 2). Testing the goodness‐of‐fit to a model of monofactorial inheritance yielded a *χ* ^2^ of 20.09 (df 9), which fails at the 5% (16.92), but not 1% probability level (21.67). We conclude that resistance to chlorpyrifos in the BZ‐R strain was a semi‐dominant autosomal character with no maternal effects which is largely but not completely controlled by one major locus.

**Table 2:** Toxicity of chlorpyrifos to the resistant strain (BZ‐R), susceptible strain (BZ‐S) and their hybrid progeny

Strain	N†	LC_50_ (mg/L) (95% FL)	Slope (SE)	RR‡	*D* §
BZ‐S (S)	125	3.6 (3.1–4.4)	3.59 (0.63)		
BZ‐R (R)	150	148 (125–174)	3.33 (0.52)	41	
F_1a_ (R♀ × S ♂)	100	36.2 (21.7–69.7)	2.86 (0.47)	10	0.24
F_1b_ (R♂ × S ♀)	120	33.2 (15.3–65.1)	3.39 (0.48)	9	0.19
F_1_ (pooled)	220	34.7 (19.4–72.6)	3.10 (0.35)	10	0.22

^†^Number of larvae tested in bioassay.

^‡^RR (resistance ratio) = 50% lethal concentration (LC_50_) (BZ‐R or F1) / LC_50_ (BZ‐S).

^§^ *D* values were calculated using the method of Stone (1968). *D* values can range from ‐1 (completely recessive) to 1 (completely dominant).

### Linkage between resistance and the A216S mutation in ace‐1

The BZ‐R strain is fixed for the S216 mutation of *ace‐1* whereas BZ‐S is fixed for A216 (Wu *et al*., 2015), so a backcross of F_1_ females from a cross between them to BZ‐S males was carried out to determine the linkage of chlorpyrifos resistance to the *ace‐1* locus (Fig. 1). PCR analysis of a control group (reared on untreated diet) of the backcross progeny yielded 12 A216/S216 heterozygotes and 11 A216 homozygotes, in good agreement with the expected 1 : 1 ratio. However the equivalent ratio among survivors of a treatment with 50 mg/L of chlorpyrifos, which produced 90.4% (226/250) mortality, was 22 heterozygotes to two A216 homozygotes, which differs significantly (*χ* ^2^ = 16.67, *P* < 0.001) from 1 : 1. These data confirm that most of resistance in BZ‐R is tightly linked to the A216S mutation of *ace‐1*.

The two homozygous A216 survivors scored may represent individuals bearing other resistance genes. Given that Wu *et al*. (2015) found BZ‐R was fixed for S216 at generation 18 when its resistance factor was only 21‐fold, some other gene(s) making a minor contribution to resistance likely had been selected for by the time its resistance factor had reached 41‐fold for the current study.

### Correlation between resistance levels and S216 frequencies in the field

**Fig. 3:**
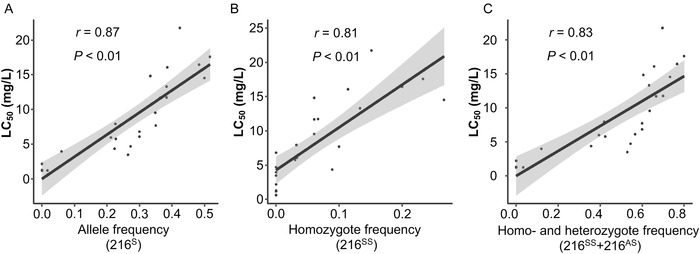
Correlations between 50% lethal concentration (LC_50_) to chlorpyrifos and S216 allele (A) and homozygote (B) and combined homo‐ and heterozygote (C) genotype frequencies in field populations of *Apolygus lucorum*. Best fit lines with their standard errors are shown.

Resistance levels to chlorpyrifos and the frequencies of the A216S mutant allele were determined in 25 field populations collected from five provinces of China during 2014–2018 (Table 3). Resistance was measured as LC_50_ as per above and a total of 838 individuals across the 25 populations were genotyped for the A216S substitution by PCR as per above. Resistance levels varied from effectively zero to about 19‐fold, and S216 allele frequencies from zero to about 52% across the 25 populations, with a strong positive correlation between the resistance factors and S216 allele and genotype frequencies (0.81 < *r* < 0.87, *P* < 0.01; Fig. 3). This confirms that the A216S mutation is a major contributor to the chlorpyrifos resistance that has evolved in field populations of *A. lucorum* from China.

**Table 3:** Resistance to chlorpyrifos and S216 allele frequency in field populations of *Apolygus lucorum*

	LC_50_ (95% CL)			Amino acids at 216			
Population	(mg/L)	RR†	N‡	A/A	A/S	S/S	S216 allele frequency (%)
SD‐BZ (2014)§	16.44 (12.24–21.88)	14.2	30	7	17	6	48.3
SD‐BZ (2015)	21.74 (16.24–31.50)	18.7	33	10	18	5	42.4
SD‐BZ (2016)	14.52 (11.18–19.68)	12.5	30	8	14	8	50.0
SD‐BZ (2017)	17.58 (13.30–25.19)	15.2	30	6	17	7	51.7
SD‐BZ (2018)	16.07 (12.27–22.16)	13.9	70	24	38	8	38.6
HB‐CZ (2014)	13.29 (10.05–18.23)	11.5	30	11	15	4	38.3
HB‐CZ (2015)	14.82 (11.69–19.06)	12.8	33	13	18	2	33.3
HB‐CZ (2016)	9.53 (6.14 –14.75)	8.2	33	12	19	2	34.8
HB‐CZ (2017)	11.67 (9.11–15.13)	10.1	33	11	20	2	36.4
HB‐CZ (2018)	11.72 (9.15–15.17)	10.1	30	9	19	2	38.3
HN‐AY (2014)	6.08 (4.54 –8.42)	5.2	30	13	16	1	30.0
HN‐AY (2015)	5.96 (4.53–7.69)	5.1	33	20	12	1	21.2
HN‐AY (2016)	5.75 (4.46 –7.37)	5.0	33	19	13	1	22.7
HN‐AY (2017)	7.92 (4.83–13.11)	6.8	31	18	12	1	22.6
HN‐AY (2018)	7.69 (5.75–10.36)	6.6	30	12	15	3	35.0
JS‐YC (2014)	6.80 (4.94–9.86)	5.9	30	12	18	0	30.0
JS‐YC (2015)	3.47 (2.73–4.31)	3.0	34	16	18	0	26.5
JS‐YC (2016)	3.94 (2.97–5.08)	3.4	50	44	6	0	6.0
JS‐YC (2017)	4.68 (3.44–6.18)	4.0	33	15	18	0	27.3
JS‐YC (2018)	4.34 (3.24–5.65)	3.7	56	36	15	5	22.3
AH‐CZ (2014)	0.60 (0.36–0.86)	0.5	30	29	1	0	1.7
AH‐CZ (2015)	2.17 (1.61‐2.87)	1.9	36	36	0	0	0
AH‐CZ (2016)	1.20 (0.76 ‐ 1.67)	1.0	30	29	1	0	1.7
AH‐CZ (2017)	1.27 (0.74‐1.85)	1.1	30	30	0	0	0
AH‐CZ (2018)	1.17 (0.76‐1.58)	1.0	30	30	0	0	0
SLF lab strain	1.16 (0.78‐1.54)	1.0	30	30	0	0	0

^†^RR (resistance ratio) = 50% lethal concentration (LC_50_) (field population)/ LC_50_ (the susceptible SLF strain).

^‡^The number of individuals genotyped.

^§^The LC_50_ data for 2014 were taken from Liu *et al*. (2015).

Given the semi‐dominant inheritance observed above, the combined frequency of the S216 homo‐ and heterozygotes may be most relevant to field exposure conditions and that would approach 75% in some of the populations, leaving limited scope for further increases in resistance due to the S216 mutation in those populations. The great majority of the variation in LC_50_ and allele and genotype frequencies observed among the 25 collections is due to differences among the five collection sites and no systematic trends over the five years of sampling are evident in the samples from any of the sites (Table 3).

Finally it is noted that the resistance factors shown in Table 3 are expressed relative to the standard laboratory line SLF, which has an LC_50_ less than half that of the susceptible BZ‐S line used in the genetic analyses above. This confirms a difference between these two susceptible strains reported by Wu *et al*. (2015) and, given that both lines are fixed for A216, it suggests that other genes making minor contributions to resistance were segregating in the parental BZ line from which BZ‐R was selected.

### Minor roles for metabolic enzymes in chlorpyrifos resistance

Bioassays in the presence of the respective synergists indicate that P450 and esterase but not GST enzymes (synergism ratios of 2.7, 3.0 and 1.2 respectively) also played some role in the chlorpyrifos resistance seen in BZ‐R (Table 4). Consistent with this finding, BZ‐R was found to have higher P450 and esterase but not GST activities than either BZ‐S or SLF (Table 5). In combination, these data suggest the minor role in chlorpyrifos resistance played by genes other than *ace‐1* in the current BZ‐R strain of *A. lucorum* may be mediated by their enhancement of P450 and esterase activities.

**Table 4:** Synergism of chlorpyrifos by PBO, DEM and DEF in three strains of *Apolygus lucorum*

Strain	Insecticide	LC_50_ (mg/L) (95%CL)	Slope ± SE	SR†
BZ‐R	Chlorpyrifos + DEF	55.5 (40.4–72.1)	2.7 ± 0.4	3.0
	Chlorpyrifos + DEM	140.0 (111.9–174.7)	3.0 ± 0.4	1.2
	Chlorpyrifos + PBO	61.8 (40.6–82.7)	2.2 ± 0.4	2.7
	Chlorpyrifos	166.7 (128.1–208.5)	3.5 ± 0.6	—
BZ‐S	Chlorpyrifos + DEF	4.0 (3.2–5.0)	3.1 ± 0.4	1.0
	Chlorpyrifos + DEM	3.5 (2.7–4.6)	2.4 ± 0.4	1.2
	Chlorpyrifos + PBO	4.2 (3.2–5.5)	2.3 ± 0.4	1.0
	Chlorpyrifos	4.1 (3.2–5.1)	2.8 ± 0.4	—
SLF	Chlorpyrifos + DEF	0.9 (0.7–1.3)	1.9 ± 0.3	1.5
	Chlorpyrifos + DEM	1.1 (0.9–1.6)	2.1 ± 0.3	1.3
	Chlorpyrifos + PBO	1.2 (0.9–1.8)	1.8 ± 0.3	1.2
	Chlorpyrifos	1.4 (0.7–2.5)	2.0 ± 0.3	—

^†^SR (synergistic ratio) = 50% lethal concentration (LC_50_) (insecticide alone) / LC_50_ (insecticide with synergist) DEF, S,S,S‐tributyl phosphorotrithioate; DEM, diethyl maleate; PBO, piperonyl butoxide

**Table 5:** Metabolic enzyme activities of three strains of *Apolygus lucorum*

				Activity ratio	
Enzyme	Substrate	Strain	Enzyme activity†	BZ‐R/SLF	BZ‐R/BZ‐S
EST (nmol/min/mg protein)	α‐naphthyl acetate	BZ‐R	90.0 ± 2.8b	2.0	1.7
		BZ‐S	53.0 ± 3.8a		
		SLF	45.3 ± 5.6a		
GST (nmol/min/mg protein)	CDNB	BZ‐R	814.3 ± 55.8a	1.1	1.2
		BZ‐S	694.5 ± 73.9a		
		SLF	764.9 ± 15.2a		
MFO (pmol/30 min/mg protein)	Ethoxycoumarin	BZ‐R	95.1 ± 7.8b	7.4	6.1
		BZ‐S	15.5 ± 0.4a		
		SLF	12.8 ± 0.4a		

^†^Means and standard errors of four biological replicates. Mean activities between strains followed by the same letter are not significantly different (analysis of variance, *P* < 0.05). CNBD, 2,4‐dinitrochlorobenzene; EST, esterase; GST, glutathione s‐transferase; MFO, mixed function oxidase.

## Discussion

Here we have demonstrated that much of the chlorpyrifos resistance in the BZ‐R strain of *A. lucorum* is tightly linked to the A216S mutation of *ace‐1* and that the resistance levels of field populations collected from China are highly correlated with S216 allele and genotype frequencies. Together with heterologous expression results from two previous studies (Wu *et al*., 2015; Zhen *et al*., 2016), there is now compelling evidence that this substitution plays a major role in chlorpyrifos resistance in both laboratory‐selected and field‐collected strains of *A. lucorum*. Given that *ace‐1* resistance mutations in other species, including direct equivalents of A216S, generally protect them against a range of OPs and carbamates (Andrews *et al*., 2004; Li & Han, 2004; Toda *et al*., 2004; Lee *et al*., 2007; Jiang *et al*., 2009; Li *et al*., 2009; Zhao *et al*., 2014), we might expect that the A216S *ace‐1* mutation in *A. lucorum* would also confer resistance to several other actives within these two insecticide classes.

Insecticide resistance conferred by insensitive acetylcholinesterases in other species can vary from semi‐recessivity to strong dominance but commonly involves semi‐dominance (Bourguet & Raymond, 1998), as seen in our case. Other examples of semi‐dominant chlorpyrifos resistance due to *ace‐1* mutations involve *Culex pipiens* (Raymond & Georghiou, 1987), *Blattela germanica* (Siegfried *et al*., 1990) and *Tetranychus urticae* (Ay & Yorulmaz, 2010). The cotton mealybug *Phenacoccus solenopsis* provides an example of semi‐recessive inheritance (Afzal *et al*., 2015). The differences likely represent quantitative differences in the underlying molecular mechanisms, for example in the relative affinities of the OP versus the natural acetylcholine substrate for binding to the acetylcholinesterase‐1 of different species.

Our previous work has estimated that the A216S substitution in *ace‐1* by itself confers about 20‐fold resistance to chlorpyrifos in *A. lucorum* (Wu *et al*., 2015). The BZ‐R strain used in the present study has 41‐fold resistance to chlorpyrifos compared with the BZ‐S strain, which suggests that one or more other genes may also make some contribution to its resistance. This concurs with evidence in both Wu *et al*. (2015) and the current study that some genes with minor effects on chlorpyrifos tolerance were indeed segregating in the original BZ line from which BZ‐R was selected. Our synergism bioassays and enzyme assays suggest that these genes could be responsible for some metabolic resistance via elevated levels of particular cytochrome P450 monooxygenase and esterase enzymes. Metabolic resistances to chlorpyrifos and other OPs mediated by either or both P450 and esterase activities have been reported in a wide variety of insects such as *A. gossypii*, *Spodoptera frugiperda* and *Tuta absoluta* (Ay & Yorulmaz, 2010; Shang *et al*., 2012; Carvalho *et al*., 2013; Edi *et al*., 2014; Haddi *et al*., 2017).

Application of chemical insecticides will remain a major component of *A. lucorum* management in China in the medium term because transgenic crops controlling mirids are not yet commercially available. The current study provides useful information and tools for designing effective resistance monitoring and management strategies for *A. lucorum* in China. We found no obvious increase in the frequencies of S216 alleles and genotypes over the five years we surveyed, which may be because mounting human toxicity concerns have seen chlorpyrifos use decline over the last few years in favor of other actives such as sulfoxaflor (Zhen *et al*., 2018). However, S216 genotype frequencies were already quite high in four of the five provinces we surveyed, ranging between 40%–50% (Henan, Jiangsu) and about 75% (Shandong, Hebei). S216 was also found, albeit at much lower frequency (less than 5%), in the fifth province we sampled (Anhui), and we found low‐level metabolic resistance as well, at least in BZ‐R. Thus chlorpyrifos, and quite likely other OPs, would seem to have limited utility in many parts of China from here on. In so much as S216 is currently responsible for most of the resistance, the PCR method we have developed could be a useful tool for ongoing monitoring work.

## Disclosure

The authors declare that they have no conflicts of interest.

## References

[ins12744-bib-0001] Afzal, M.B.S. , Ijaz, M. , Farooq, Z. , Shad, S.A. and Abbas, N. (2015) Genetics and preliminary mechanism of chlorpyrifos resistance in *Phenacoccus solenopsis* Tinsley (Homoptera: Pseudococcidae). Pesticide Biochemistry and Physiology, 119, 42–47. 25868815 10.1016/j.pestbp.2015.02.008

[ins12744-bib-0002] Andrews, M.C. , Callaghan, A. , Field, L.M. , Williamson, M.S. and Moores, G.D. (2004) Identification of mutations conferring insecticide insensitive AChE in the cotton‐melon aphid, *Aphis gossypii* Glover. Insect Molecular Biology, 13, 555–561. 15373812 10.1111/j.0962-1075.2004.00517.x

[ins12744-bib-0003] Ay, R. and Yorulmaz, S. (2010) Inheritance and detoxification enzyme levels in *Tetranychus urticae* Koch (Acari: Tetranychidae) strain selected with chlorpyrifos. Journal of Pest Science, 83, 85–93.

[ins12744-bib-0004] Bergé, J.B. and Ricroch, A.E. (2010) Emergence of minor pests becoming major pests in GE cotton in China: What are the reasons? What are the alternative practices to this change of status? GM Crops, 4, 214–219. 10.4161/gmcr.1.4.13421 21844676

[ins12744-bib-0005] Bourguet, D. and Raymond, M. (1998) The molecular basis of dominance relationships: the case of some recent adaptive genes. Journal of Evolutionary Biology, 11, 103–122.

[ins12744-bib-0007] Carvalho, R.A. , Omoto, C. , Field, L.M. , Williamson, M.S. , Bass, C. (2013) Investigating the molecular mechanisms of organophosphate and pyrethroid resistance in the fall armyworm *Spodoptera frugiperda* . PLoS ONE, 8, e62268. 23614047 10.1371/journal.pone.0062268 PMC3629120

[ins12744-bib-0008] Edi, C.V. , Djogbénou, L. , Jenkins, A.M. , Regna, K. , Muskavitch, M.A.T. , Poupardin, R . *et al* (2014) CYP6 P450 enzymes and *ACE‐1* duplication produce extreme and multiple insecticide resistance in the malaria mosquito *Anopheles gambiae* . PLoS Genetics, 10, e1004236. 24651294 10.1371/journal.pgen.1004236 PMC3961184

[ins12744-bib-0009] Georghiou, G.P. (1969) Genetics of resistance to insecticides in houseflies and mosquitoes. Experimental Parasitology, 26, 224–255. 4927150 10.1016/0014-4894(69)90116-7

[ins12744-bib-0010] Haddi, K. , Berger, M. , Bielza, P. , Rapisarda, C. , Williamson, M.S. , Moores, G . *et al* (2017) Mutation in the ace‐1 gene of the tomato leaf miner (*Tuta absoluta*) associated with organophosphates resistance. Journal of Applied Entomology, 141, 612–619.

[ins12744-bib-0011] Jiang, X.J. , Qu, M.J. , Denholm, I. , Fang, J.C. , Jiang, W.H. , Han, Z.J. (2009) Mutation in acetylcholinesterase1 associated with triazophos resistance in rice stem borer, *Chilo suppressalis* (Lepidoptera: Pyralidae). Biochemical and Biophysical Research Communications, 378, 269–272. 19028456 10.1016/j.bbrc.2008.11.046

[ins12744-bib-0012] Lee, D.W. , Choi, J.Y. , Kim, W.T. , Je, Y.H. , Song, J.T. , Chung, B.K . *et al* (2007) Mutations of acetylcholinesterase1 contribute to prothiofos resistance in *Plutella xylostella* (L.). Biochemical and Biophysical Research Communicationns, 353, 591–597. 10.1016/j.bbrc.2006.12.088 17196934

[ins12744-bib-0013] Li, C. , Dong, Y. , Zhang, X. and Zhao, T. (2009) An amino acid substitution on the acetylcholinesterase in the field strains of house mosquito, *Culex pipiens pallens* (Diptera: Culicidae) in China. Entomological News, 120, 464–475.

[ins12744-bib-0014] Li, F. and Han, Z.J. (2004) Mutations in acetylcholine sterase associated with insecticide resistance in the cotton aphid, *Aphis gossypii* Glover. Insect Biochemistry and Molecular Biology, 34, 397–405. 15041023 10.1016/j.ibmb.2004.02.001

[ins12744-bib-0015] Liu, J. , Li, T.T. , Huang, J.M. , Kang, Z.K. , Yang, Y.H. , Wu, Y.D . *et al* (2015) Resistance to beta‐cypermethrin and chlorpyrifos in populations of *Apolygus lucorum* from the Yellow and Changjiang river cotton growing areas of China. Chinese Journal of Applied Entomolology, 52, 616–622.

[ins12744-bib-0016] Lu, Y.H. , Qiu, F. , Feng, H.Q. , Li, H.B. , Yang, Z.C. , Wyckhuys, K.A.G . *et al* (2008) Species composition and seasonal abundance of pestiferous plant bugs (Hemiptera: Miridae) on Bt cotton in China. Crop Protection, 27, 465–472.

[ins12744-bib-0017] Lu, Y.H. , Wu, K.M. , Jiang, Y.Y. , Xia, B. , Li, P. , Feng, H.Q . *et al* (2010) Mirid bug outbreaks in multiple crops correlated with wide‐scale adoption of Bt cotton in China. Science, 328, 1151–1154. 20466880 10.1126/science.1187881

[ins12744-bib-0018] Oakeshott, J.G. , Devonshire, A.D. , Claudianos, C. , Sutherland, T.D. , Horne, I. , Campbell, P.M . *et al* (2005) Comparing the organophosphorus and carbamate insecticide resistance mutations in cholin‐ and carboxylesterases. Chemico‐Biological Interactions, 157, 269–275. 16289012 10.1016/j.cbi.2005.10.041

[ins12744-bib-0020] Raymond, M. , Pasteur, N. and Georghiou, G.P. (1987) Inheritance of chlorpyrifos resistance in *Culex pipiens* L. (Diptera: Culicidae) and estimation of the number of genes involved. Heredity, 58, 351–356.

[ins12744-bib-0021] Russell, R.J. , Claudianos, C. , Campbell, P.M. , Horne, I. , Sutherland, T.D. and Oakeshott, J.G. (2004) Two major classes of target site insensitivity mutations confer resistance to organophosphate and carbamate insecticides. Pesticide Biochemistry and Physiology, 79, 84–93.

[ins12744-bib-0023] Shang, Q. , Pan, Y. , Fang, K. , Xi, J. and Brennan, J.A. (2012) Biochemical characterization of acetylcholinesterase, cytochrome P450 and cross‐resistance in an omethoate‐resistant strain of *Aphis gossypii* Glover. Crop Protection, 31, 15–20.

[ins12744-bib-0024] Siegfried, B.D. , Scott, J.G. , Roush, R.T. and Zeichner, B.C. (1990) Biochemistry and genetics of chlorpyrifos resistance in the German cockroach, *Blattella germanica* (L). Pesticide Biochemistry and Physiology, 38, 110–121.

[ins12744-bib-0025] Snodgrass, G.L. (1996) Glass‐vial bioassay to estimate insecticide resistance in adult tarnished plant bugs (Heteroptera: Miridae). Journal of Economic Entomology, 89, 1053–1059.

[ins12744-bib-0026] Stone, B. (1968) A formula for determining degree of dominance in cases of monofactorial inheritance of resistance to chemicals. Bulletin of the World Health Organization, 38, 325–326. 5302309 PMC2554319

[ins12744-bib-0027] Tabashnik, B.E. (1991) Determining the mode of inheritance of pesticide resistance with backcross experiments. Journal of Economic Entomology, 84, 703–712. 1885840 10.1093/jee/84.3.703

[ins12744-bib-0028] Toda, S. , Komazaki, S. , Tomita, T. and Kono, Y. (2004) Two amino acid substitutions in acetylcholinesterase associated with pirimicarb and organophosphorous insecticide resistance in cotton aphid, *Aphis gossypii* Glover (Homoptera: Aphididae). Insect Molecular Biology, 13, 549–553. 15373811 10.1111/j.0962-1075.2004.00513.x

[ins12744-bib-0029] Wheeler Jr, A.G. (2001) Biology of the Plant Bugs (Hemiptera: Miridae): Pests, Predators, Opportunists. pp. 528 Cornell University Press, Ithaca, NY, USA.

[ins12744-bib-0030] Wu, K.M. , Lu, Y.H. , Feng, H.Q. , Jiang, Y.Y. and Zhao, J.Z. (2008) Suppression of cotton bollworm in multiple crops in China in areas with Bt toxin‐containing cotton. Science, 321, 1676–1678. 18801998 10.1126/science.1160550

[ins12744-bib-0031] Wu, S.W. , Zuo, K.R. , Kang, Z.K. , Yang, Y.H. , Oakeshott, J.G. and Wu, Y.D. (2015) A pointmutation in the acetylcholinesterase‐1 gene is associated with chlorpyrifos resistance in the plant bug *Apolygus lucorum* . Insect Biochemistry and Molecular Biology, 65, 75–82. 26363297 10.1016/j.ibmb.2015.09.005

[ins12744-bib-0032] Zhao, M.H. , Dong, Y. , Ran, X. , Wu, Z.M. , Guo, X.X. , Zhang, Y.M . *et al* (2014) Point mutations associated with organophosphate and carbamate resistance in Chinese strains of *Culex pipiens quinquefasciatus* (Diptera: Culicidae). PLoS ONE, 9, e95260. 24788312 10.1371/journal.pone.0095260 PMC4006752

[ins12744-bib-0033] Zhen, C.A. , Miao, L. , Liang, P. and Gao, X.W. (2016) Survey of organophosphate resistance and an A1a216Ser substitution of acetylcholinesterase‐1 gene associated with chlorpyrifos resistance in *Apolygus lucorum* (Meyer‐Dür) collected from the transgenic Bt cotton fields in China. Pesticide Biochemistry and Physiology, 132, 29–37. 27521910 10.1016/j.pestbp.2016.04.008

[ins12744-bib-0034] Zhen, C.A. and Gao, X.W. 2016 A point mutation (L1015F) of the voltage‐sensitive sodium channel gene associated with lambda‐cyhalothrin resistance in *Apolygus lucorum* (Meyer‐Dür) population from the transgenic Bt cotton field of China. Pesticide Biochemistry and Physiology, 127, 82–89. 26821662 10.1016/j.pestbp.2015.09.011

[ins12744-bib-0035] Zhen, C.A. , Miao, L. and Gao, X.W. (2018) Sublethal effects of sulfoxaflor on biological characteristics and vitellogenin gene (*AlVg*) expression in the mirid bug, *Apolygus lucorum* (Meyer‐Dür). Pesticide Biochemistry and Physiology, 144, 57–63. 29463409 10.1016/j.pestbp.2017.11.008

